# Research on the Emotions Based on Brain*-*Computer Technology: A Bibliometric Analysis and Research Agenda

**DOI:** 10.3389/fpsyg.2021.771591

**Published:** 2021-11-01

**Authors:** Wei Yan, Xiaoju Liu, Biaoan Shan, Xiangxian Zhang, Yi Pu

**Affiliations:** School of Management, Jilin University, Changchun, China

**Keywords:** emotion, brain-computer, digital technology, bibliometric analysis, research agenda

## Abstract

This study conducts a scientific analysis of 249 literature on the application of brain-computer technology in emotion research. We find that existing researches mainly focus on engineering, computer science, neurosciences neurology and psychology. PR China, United States, and Germany have the largest number of publications. Authors can be divided into four groups: real-time functional magnetic resonance imaging (rtfMRI) research group, brain-computer interface (BCI) impact factors analysis group, brain-computer music interfacing (BCMI) group, and user status research group. Clustering results can be divided into five categories, including external stimulus and event-related potential (ERP), electroencephalography (EEG), and information collection, support vector machine (SVM) and information processing, deep learning and emotion recognition, neurofeedback, and self-regulation. Based on prior researches, this study points out that individual differences, privacy risk, the extended study of BCI application scenarios and others deserve further research.

## Introduction

In the area of organizational psychology and individual psychology, human emotion is an essential research topic and is concerning by many scholars. The definition of emotion is not widely agreed among scholars. According to existing researches, emotion consists of various structures (including joy, love, shame, affect, mood), and could be divided into different dimensions, e.g., approach-oriented or withdrawal-oriented emotion, pleasure or arousal emotion, and self-caused or other-caused emotion, basic or social emotion (e.g., Ashforth and Humphrey, 1995; Butt et al., 2005). It is considered as a key antecedent of cognition and perception, and strongly influences behaviors of actors.

Prior studies in this area have focused on two aspects. Firstly, the conceptual study of emotions, including definition, dimension, and measurement (e.g., Ashforth and Humphrey, 1995; Barrett et al., 2007). Secondly, some literature has revealed that emotions play important roles in organizational and individual behaviors, including decision making, negotiation strategy, work performance, innovation, and entrepreneurship (e.g., Ashforth and Humphrey, 1995; Butt et al., 2005; Roundy, 2014). Besides, scholars have analyzed emotions of individuals from various organizations, such as companies, social organizations, hospitals. These studies have significantly improved the research field of psychology and have highly contributed to the understanding of emotional process.

Recently, the progress of digital technologies is promoting the development of emotion research. The rapid development of emerging digital technologies such as artificial intelligence (AI), virtual reality, human-computer interaction, and machine learning has enhanced their application in psychology (Shan et al., 2021). Especially, researches on human emotion states via brain-computer technology have increased dramatically in the past several years. However, prior researches mainly explore the concept of emotions and their influences on individuals or organizations. We still know little about how to analyze emotions by using advanced technologies.

Especially, brain-computer technology strongly enhances the ability to recognize human emotion. It provides a new tool for traditional emotion research. The emotion analysis via brain-computer technology plays a key role in designing communication systems (Atkinson and Campos, 2016). Considering that the study on this area is still in its infancy stage, it is not surprising that we know little about the research contents and progress in this field. As Wang et al. (2014) pointed out, the relationship between emotion and new technology, such as emotion recognition based on BCI, is an important subject that needs to be developed. In order to address this gap, this study reviews literatures on emotions based on brain-computer technology. We try to systematically introduce existing researches in this area and provide future research directions.

The contribution of this study mainly includes the following two aspects. On the one hand, we use scientific bibliometric analysis method to visually analyze the researches in the field of affective BCI. We use VOSviewer software to build a research topic framework to help researchers have a more comprehensive understanding of the field. On the other hand, in the part of future research, we point out the gaps in existing research and possible advanced research directions, laying a foundation for subsequent research.

This study is structured as follows. Firstly, the process of methodology is introduced. And then, analysis results of publication years, research areas, countries, authors, and cooperation networks, and the most cited papers are provided. Follow that, theme clustering analysis is completed and relevant results are produced. Finally, future researches and conclusions are provided.

## Methodology

We study the application of BCI in the field of emotion research through using the literature bibliometric technology being widely used increasingly and choosing the bibliometric software VOSviewer. Furthermore, we also try to explore the publication situation, the distribution of research fields and intellectual networks, the most influential publications, existing research streams, and future research directions of this topic in recent years, to promote further research and attention of academia on this research topic. Bibliometric technology is a quantitative method based on bibliometric data. Compared with traditional literature analysis methods, it is more systematic and transparent (Wörfel, 2019), and is more suitable for analyzing the distribution of intellectual networks (Velt et al., 2020), structure and dynamic trends (Baier-Fuentes et al., 2019), focus and future research directions (Zaheer et al., 2019), and others of a given research field. Specific process of bibliometric analysis is as follows.

### Question Formulation

Our research aims to comprehensively demonstrate current academic researches on the application of BCI in the field of emotion research through a bibliometric analysis of existing related publications. We try to discuss and solve following research questions:

• How has the academia’s attention to this research topic changed in recent years?
• How are the research areas of existing researches distributed?
• Which countries are most interested in this topic?
• Who are the most productive authors in this topic?
• What are the most influential publications about this topic?
• What types of existing researches about this topic can be divided into? What are the foci of each category?
• What are the possible future research directions about this topic?

### Literature Selection

To determine publications most relevant to the application of brain computer interaction in the field of emotion research, we first use *((TS* = *(“emotion^∗^” and “brain-computer^∗^”)) AND LA* = *(English)) AND DT* = *(Article)* as a search term to retrieve in the Core Collection Database of Web of Science, the most commonly used database in bibliometrics. English is chosen to ensure the quality and understandability of publications, and peer-reviewed articles are chosen to ensure the scientificity and authority of publications. And 270 publications are obtained (the retrieval was conducted on August 5, 2021). Next, to eliminate publications that are not related to our research topic (e.g., art, physics), research team members read title, abstract, author keywords, and keywords plus^®^ of these publications, respectively, and further read introduction or full text when necessary. After integrating opinions of various researchers, we finally get 249 publications as our research objects.

### Bibliometric Analysis

With the help of the Web of Science database and VOSviewer software, we conduct a bibliometric analysis on the selected literature set. We first analyze publication years, research areas, countries, authors, and cooperation networks, and most cited papers in BCI and emotion research. Through these analyses, scholars and relevant researchers can have a clearer understanding of the research status of BCI and emotion research, so that they can find research questions they are interested in.

### Thematic Clustering Analysis

To identify research clusters of our research topic and find out current foci and future research directions, we continue to use VOSviewer software to conduct thematic clustering analysis on selected publications. This part will be discussed separately.

## Results

### Publication Years

In 2005, Pun et al. (2006) shared a paper titled *Brain-Computer Interaction Research at The Computer Vision and Multimedia Laboratory* on 3rd International Meeting on Brain-Computer Interface Technology in University of Geneva. This paper is related to the work of the Multi-modal Interaction Group at the University of Geneva in the field of BCI. The team has three main research topics: brain source activity recognition, BCI protocol and performance research, and assessment of users’ emotion states. This paper first focused on the application of BCI in emotion field and was published in IEEE Transactions on Neural Systems and Rehabilitation Engineering in 2006.

**FIGURE 1:**
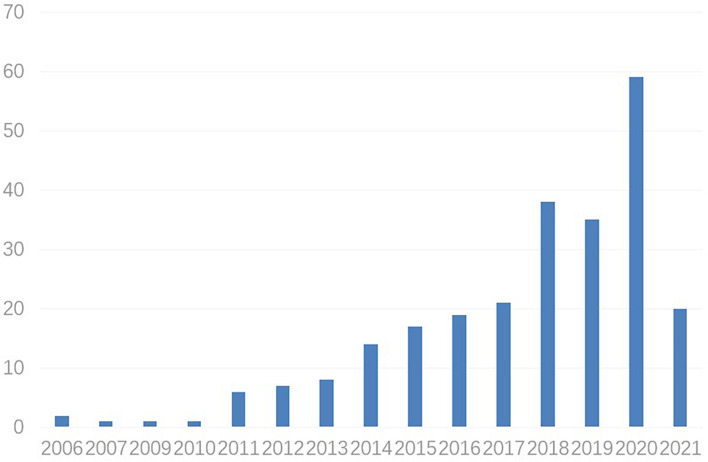
Number of documents per year in BCI and emotions research.

In recent years, scholars have paid more and more attention to the application of BCI in emotion field. In this study, the number of annual publications about this topic is counted (Figure 1). In 2006, the first paper on the application of BCI in emotion field was published, but it failed to attract widespread attention of scholars. In 2011, 7 related articles were published successively, exceeding the total number of articles published in the past 5 years. The progress of BCI technology has greatly promoted research interests of scholars. The number of articles published in 2018 reached 38, about twice the number published in 2017. With the increase in the number of applied studies of BCI in emotion field, it has become a very important work to sort out and analyze these documents with the help of scientific software systems.

### Research Areas

**FIGURE 2:**
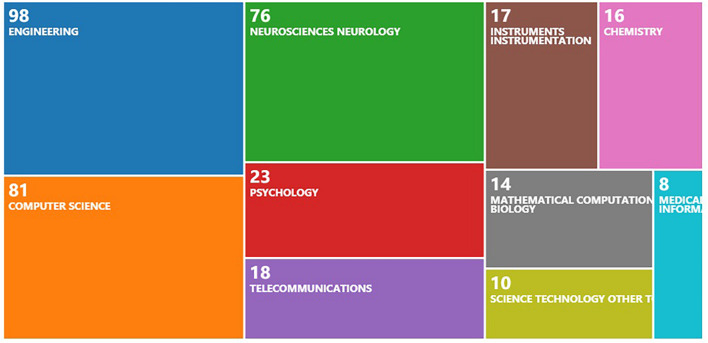
Number of documents by research areas in BCI and emotions research.

Figure 2 shows the research field distribution of the 249 articles we retrieved. The same article may belong to multiple fields at the same time. The top three areas of this study are Engineering, Computer Science, and Neurosciences Neurology, respectively with 98, 81, and 76 papers. This is because BCI is an interdisciplinary research field involving multiple disciplines including engineering, computer science, and neuroscience, aiming to study and develop the ability to identify, interpret, process, and model human brain activity. Human emotion recognition is closely related to Neurosciences Neurology and Psychology, which have laid the foundation for the application of BCI in the identification of human emotions.

### Countries

**FIGURE 3:**
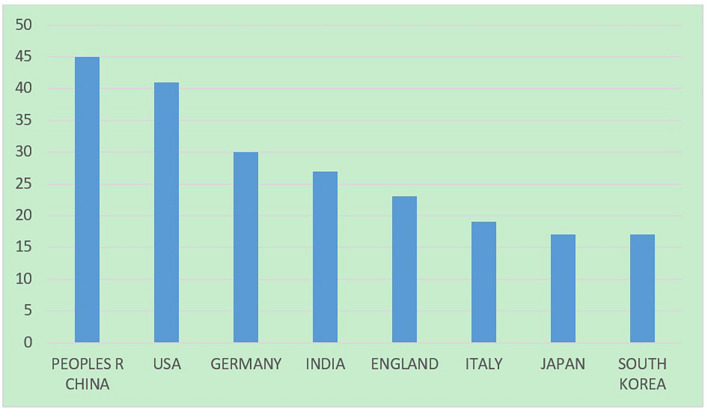
Number of documents by countries in BCI and emotions research.

In order to understand the attention of each country on BCI and emotion research, the number of published papers in each country is statistically analyzed (Figure 3). Countries with more publications are more interested in the topic. Peoples R China ranks first with 45 articles, accounting for 18.072% of the total articles, followed by the United States with 41 articles and Germany with 30 articles. In addition, India, England, Italy, Japan, and South Korea also have good research results.

### Authors and Cooperation Networks

The following is a study on the number of authors’ publications (Table 1). Birbaumer is the most productive scholar in the field of affective BCI, with 10 articles published from 2007 to 2017, accounting for 4.016% of the total literature. Sitaram, Bajaj, Cichocki, Kubler, Placidi, and Spezialetti are prolific authors in the field of emotion research based on BCI, with the first two authors publishing 7 and 6 articles, respectively, and the remaining authors publishing 5 articles.

**TABLE 1:** The most productive authors in BCI and emotion research.

	Authors	Record count	% of 249	Typical article
1	Birbaumer N	10	4.016	Regulation of anterior insular cortex activity using real-time fMRI
2	Sitaram R	7	2.811	Regulation of anterior insular cortex activity using real-time fMRI
3	Bajaj V	6	2.41	Emotion recognition from single-channel EEG signals using a two-stage correlation and instantaneous frequency-based filtering method
4	Cichocki A	5	2.008	The changing face of P300 BCIs: A Comparison of stimulus changes in a P300 BCI involving faces, emotion, and movement
4	Kubler A	5	2.008	The Changing face of P300 BCIs: A comparison of stimulus changes in a P300 BCI involving faces, emotion, and movement
4	Placidi G	5	2.008	A real-time classification algorithm for EEG-based BCI driven by self-induced emotions
4	Spezialetti M	5	2.008	A real-time classification algorithm for EEG-based BCI driven by self-induced emotions
7	Chau T	4	1.606	Classification of prefrontal activity due to mental arithmetic and music imagery using hidden Markov models and frequency domain near-infrared spectroscopy
7	Daly I	4	1.606	Affective brain-computer music interfacing
7	Falk TH	4	1.606	Classification of prefrontal activity due to mental arithmetic and music imagery using hidden Markov models and frequency domain near-infrared spectroscopy

**FIGURE 4:**
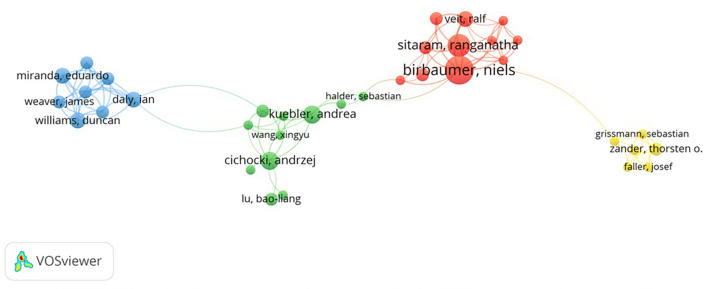
Authors cooperation networks in BCI and emotions research.

Further, in the field of BCI and emotion research, scholars have carried out extensive cooperation. A total of 869 authors has co-published 249 articles. Only authors who have published more than two articles in collaboration with others will be included in the collaboration network. In this way, 125 eligible authors are selected. Then we chose the largest set of 36 authors for our study because it is the most widely collaborative network of authors. VOSviewer classifies authors according to the authors’ cooperation, and colors the different groups. The collaboration network is shown in Figure 4. Authors are divided into four categories.

### Group 1: Real-Time Functional Magnetic Resonance Imaging Research Group

Authors in the first category in Figure 4 are in red group, include Niels Birbaumer, Ranganatha Sitaram, Ralf Veit, Andrea Caria, Antonino Raffone, etc. By reading articles of the first type authors, we find that they pay more attention to the effect of functional magnetic resonance imaging (fMRI) on the insula state. In 2007, Caria et al. (2007) used real-time functional magnetic resonance imaging (rtfMRI) for the first time to train and study the willpower control of brain regions related to emotion and confirmed that rtfMRI may enable local brain activity to achieve self-regulation. Distinguishing brain response to different stimuli is a prerequisite for communication through BCI. In the context of fMRI, Van der Heiden et al. (2014) found that insula and inferior frontal triangularis activations could distinguish brain responses under different emotional sound stimuli, which laid a foundation for the applied research of BCI. In addition, researchers have investigated the volitional control of the anterior insula in criminal psychopaths (Sitaram et al., 2014) and the application of BCI in Alzheimer’s disease patients (Liberati et al., 2012). According to their research content, we name the first group rtfMRI Research Group.

### Group 2: Brain-Computer Interface Impact Factors Analysis Group

We refer to the green group of authors as the BCI Impact Factors Analysis Group, including Andrzej Cichocki, Andrea Kuebler, Xingyu Wang, Baoliang Lu, etc. Above authors focused on the effects of emotional face, background music, and emotion recognition pathways on BCI performance. Bakardjian et al. (2011) found that emotional faces can enhance the steady-state visual responses for BCI and improve the reliability of results. Zhou et al. (2016) explored the influence of background music on BCI performance when users used auditory BCI. The study showed that background music would not affect the effect of auditory BCI, but it is recommended to add background music when designing auditory BCI since users preferred BCI with it. Combining brain waves and eye movements, Zheng et al. (2019) proposed a multi-modal emotion recognition framework named EmotionMeter, which was better than the single mode for human emotion recognition. Studies have found that brain waves are good at identifying happy emotions, while eye movements have advantages in identifying fear emotions.

### Group 3: Brain-Computer Music Interfacing Group

In the blue group, authors conducted a series of studies on brain-computer music interfacing (BCMI). Therefore, this study names it BCMI group, including Eduardo Miranda, Ian Daly, James Weaver, Duncan Williams, Asad Malik, etc. Eaton et al. (2015) proposed a multi-user affective BCMI for performers and audiences to measure their emotional changes in concert scene. The multi-user affective BCMI provides a platform for personalized design of music by matching music and emotion. Daly et al. (2016) developed an affective brain-computer music interfacing that can regulate users’ emotions, which has the potential to be used in music therapy and entertainment. Daly et al. (2020) further proposed a personalized method that could be used for BCMI emotional state detection. Compared with population-based detection method, the accuracy of personalized emotion detection was significantly improved.

### Group 4: User Status Research Group

Sebastian Grissmann, Josef Pieces, Thorsten O Zander, Peter Gerjets, Martin Spueler et al., shown in the yellow group in Figure 4, belong to the fourth category of authors. This group’s name is the User Status Research Group. Zander and Kothe (2011) proposed passive BCI, which is an extension method of BCI. It integrates cognitive detection into BCI technology and provides valuable information such as users’ emotional intentions and states for the technical system. Grissmann et al. (2017) argued that the previous BCI approach focused on detecting a single aspect of user states in EEG was limited and we also need to consider the effect of unknown mental states on BCI performance. It is found that adding user state information can improve the application of BCI in real-world scenarios. Grissmann et al. (2020) believed that when BCI was applied in actual situation, user state recognition would be affected by users’ psychological states. Their results showed that when researchers classified working memory load under affective valence, the classification accuracy was significantly improved.

In this paper, authors and their cooperation networks are classified, and the main research contents of each group are analyzed in detail. The main purpose of this part is to help beginners find articles to study and to provide a guide for beginners in the field. For example, if beginners are interested in the study of affective BCMI, they can carry out a targeted study on articles of BCMI Group.

### The Most Cited Papers

The number of citations of an article can reflect the recognition degree of the academic community to this article. The greater the total citation number, the more classic and influential the article. Table 2 lists the top 10 articles with the most cited numbers in the affective BCI field. *Toward passive brain-computer interfaces: applying brain-computer interface technology to human-machine systems in general* was cited 369 times, ranking first in Table 2. This article was published by Zander and Kothe in 2011 on Iop Publishing Ltd. This paper focused on the study of passive BCI, which is a BCI extension method integrating BCI and cognitive monitoring. Through reading these 10 articles, we find that these articles mainly study the regulation of brain activity by real-time fMRI, the classification of EEG data by machine learning and the identification of emotions according to EEG and so on. These articles laid a foundation for subsequent research on affective BCI.

**TABLE 2:** The most cited papers in BCI and emotion research.

	Article title	Authors	Year	Publisher	Time cited
1	Toward passive brain-computer interfaces: applying brain-computer interface technology to human-machine systems in general	Zander, Thorsten O.; Kothe, Christian	2011	Iop Publishing Ltd.	369
2	Emotional state classification from EEG data using machine learning approach	Wang, Xiao-Wei; Nie, Dan; Lu, Bao-Liang	2014	Elsevier	277
3	Regulation of anterior insular cortex activity using real-time fMRI	Caria, Andrea; Veit, Ralf; Sitaram, Ranganatha et al.	2007	Academic Press Inc., Elsevier Science	234
4	Improving BCI-based emotion recognition by combining EEG feature selection and kernel classifiers	Atkinson, John; Campos, Daniel	2016	Pergamon-Elsevier Science Ltd.	148
5	Toward an EEG-based recognition of music liking using time-frequency analysis	Hadjidimitriou, Stelios K.; Hadjileontiadis, Leontios J.	2012	Ieee-Inst Electrical Electronics Engineers Inc.	114
6	Brain-computer interface-based communication in the completely locked-in state	Chaudhary, Ujwal; Xia, Bin; Silvoni, Stefano et al.	2017	Public Library Science	109
7	Classification of prefrontal activity due to mental arithmetic and music imagery using hidden Markov models and frequency domain near-infrared spectroscopy	Power, Sarah D.; Falk, Tiago H.; Chau, Tom	2010	Iop Publishing Ltd.	104
8	Single-trial classification of NIRS signals during emotional induction tasks: toward a corporeal machine interface	Tai, Kelly; Chau, Tom	2009	Bmc	94
9	Identifying stable patterns over time for emotion recognition from EEG	Zheng, Wei-Long; Zhu, Jia-Yi; Lu, Bao-Liang	2019	Ieee-Inst Electrical Electronics Engineers Inc.	91
10	The changing face of P300 BCIs: A comparison of stimulus changes in a P300 BCI involving faces, emotion, and movement	Jin, Jing; Allison, Brendan Z.; Kaufmann, Tobias et al.	2012	Public Library Science	81

## Theme Clustering Analysis

**FIGURE 5:**
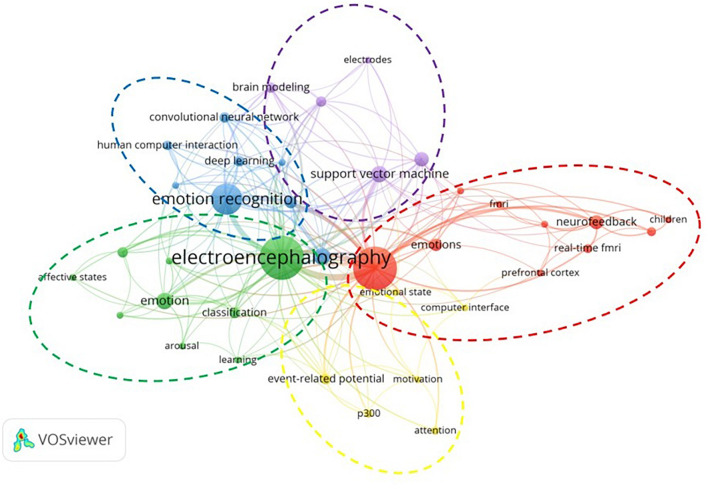
Keywords co-occurrence analysis in BCI and emotions research.

Keywords can help readers quickly understand the content of an article. With the help of VOSviewer software, the co-occurrence analysis of keywords in 249 literatures is carried out. The classification of articles according to keywords helps us construct the framework of BCI applied research in emotion field. In 249 literatures, there are 791 author keywords. We limit keyword occurrence to at least 3 times and finally obtain 58 keywords for keyword co-occurrence analysis. In order to get accurate classification results, we also combine synonymous keywords. For example, we replace “brain computer interfaces,” “brain-computer interface,” and “brain-computer interfaces” with “brain computer interface.” The clustering results of keywords are shown in Figure 5. From Figure 5, we can intuitively find that brain computer interface, electroencephalography, and emotion recognition are keywords appeared most frequently, which are related to our literature screening method and also reflect research topics in this field. Specifically, the study of the affective BCI field can be divided into five parts.

### Cluster 1: External Stimulus and Event-Related Potential

The yellow part in Figure 5 is cluster 1, which is referred to as external stimulus and event-related potential (ERP). Keywords in cluster 1 include event-related potential, p300, attention, computer interface, emotional state, motivation, etc.

ERP is a special kind of brain evoked potential, also known as “cognitive potential.” ERP reflects electrical activities of our brain in the cognitive process, and is a psychological response to external stimuli (such as vision, hearing, touch, etc.). Classic ERP mainly includes P1, N1, P2, N2, P300, N400, etc. P300 is one of the most used evoked potentials for BCI. P300 is an endogenous component that is not affected by physical properties of the stimulus, and is related to cognitive functions such as attention, recognition and memory (Polich, 2007).

Cluster 1 focuses on external stimulus and cognitive potential, especially on external stimulus and P300. P300-based BCI can be used to identify the influence of external stimulus on the tester’s emotional state, and can accurately identify the focus of attention of the tester. Pan et al. (2018) developed a BCI system for emotion recognition in patients with consciousness disorders. Subjects received a random stimulus to evoke the P300 potential. BCI monitors P300 potential and feedback results in time. The results show that the BCI system can be used to conduct cognitive experiments on patients with consciousness disorders. Ganin et al. (2019) took patients with anorexia nervosa as the research object and observed their EEG responses when they received different emotional stimuli. The study found that images of thin people’s body parts caused higher ERP amplitudes than images of food. The study also confirmed that P300-based BCI can effectively identify covert emotional foci of attention. Kleih and Kubler (2013) focused on effects of motivation for helping and empathy on P300-BCI performance. It was found that helping motivation did not affect P300 amplitude and BCI performance. However, the P300 amplitudes of high empathy testers were significantly lower than those of low empathy testers. The reason may be that people with high empathy are better able to understand external emotional stimuli and get more emotionally involved. As a result, their attention assigned to BCI tasks was reduced.

### Cluster 2: Electroencephalography and Information Collection

Cluster 2 (green) mainly consists of electroencephalography, affective brain-computer interface, affective states, arousal, classification, cognition, emotion, learning, transfer learning, etc. By reading corresponding articles, we find that cluster 2 research is related to electroencephalography (EEG) and information collection. Therefore, we call the cluster 2 EEG and information collection.

BCI records EEG signals, which are then processed and analyzed in a series of ways, and then converted into output signals that can be controlled by external devices. EEG is signal recording changes of scalp potential, which reflects the activity of the cerebral cortex to a certain extent. It has advantages of low cost, simple collection and high time resolution (Alarcao and Fonseca, 2019). The decoding of EEG signal is the key technology for BCI.

Cluster 2 revolves around EEG and information collection. Specifically, Cluster 2 focuses on how EEG signals are collected and analyzed when using emotional brain-computer interfaces. Scholars agree that emotional changes are closely linked to electrical signals in brain. Mowla et al. (2020) used laboratory data and DEAP database to evaluate the effectiveness of EEG recordings in identifying emotional states. Atkinson and Campos (2016) proposed a feature-based emotion recognition model for EEG and confirmed the effectiveness of the model. Charland et al. (2015) believed that emotion recognition should not be limited to EEG, but should collect data information through multiple channels. Therefore, authors proposed a method for collecting and synchronizing multidimensional data to simultaneously analyze electroencephalography, electrodermal, eye-tracking, and facial emotion recognition data. Lin and Jung (2017) also focused on the application of transfer learning in EEG emotion classification system. They proposed that conditional transfer learning framework can effectively improve the classification of individual default emotions.

### Cluster 3: Support Vector Machine and Information Processing

When exploring the third group support vector machine (SVM) and information processing cluster (purple), there are items like SVM, feature extraction, machine learning, brain modeling, electrodes, etc.

SVM classifies data in binary mode according to supervised learning. It is a machine learning method to solve classification problems and an important tool to solve data classification (Cortes and Vapnik, 1995; Sexton et al., 2017).

Cluster 3 focuses on SVM and information processing. Identifying emotions from EEG signals can help people with emotional expression disorders to better communicate with people and environment around them. Emotion recognition requires information processing of EEG signals. Information processing includes information preprocessing, feature extraction and selection, and emotion classification. Khare and Bajaj (2021) believed that for multi-components EEG signal, it is necessary to decompose it into component sets before extracting hidden information. Authors also proposed optimized variational mode decomposition. The method uses the eigenvector centrality method to select dominant channels. Arpaia et al. (2020) proposed a wearable single-channel instrument that can monitor human stress states by analyzing changes of EEG signals in real time. This instrument evaluates EEG pressure features by analyzing the frontal asymmetry and uses machine learning classifiers such as SVM to classify EEG, with high classification accuracy. Gannouni et al. (2020) proposed VAD model to extract, select and classify EEG signals from three dimensions of valence, arousal, and dominance, respectively.

### Cluster 4: Deep Learning and Emotion Recognition

Deep learning and emotion recognition cluster (blue) dominate by keywords such as emotion recognition, emotion classification, deep learning, affective computing, convolutional neural network (CNN), human computer interaction, medical signal processing, signal processing, etc.

Deep learning is a deep machine learning model, which is often used in a variety of supervised pattern recognition problems. Deep learning mainly includes CNN (Lecun et al., 1998), deep belief nets (DBN) (Hinton et al., 2006), recurrent neural network (RNN) (Jieun et al., 2020), etc. Different types of deep learning apply to different scenarios. For example, CNN is mostly used in image processing, and RNN is often used in natural language processing.

Cluster 4 focuses on deep learning and emotion recognition, especially on CNN and emotion recognition. CNN is a feedforward neural network, which carries out convolution and pooling operations on the basis of the neural network. CNN is widely used in the field of emotion recognition. Chen et al. (2021) proposed data augmentation method borderline-synthetic minority oversampling technique based on CNN. Firstly, EEG signals were preprocessed, and then frequency domain features were extracted by data enhancement algorithm. Finally, the valence and arousal dimensions of emotions were classified by CNN. Hwang et al. (2020) proposed a new emotion recognition method using CNN, which can ensure the comprehensiveness of information. Authors test the method on SEED data set, and their results show that the method is effective and accurate. Li et al. (2018) believed that traditional machine learning has the problem of overfitting in the process of emotion recognition based on EEG. Therefore, they proposed to use hierarchical convolutional neural network (HCNN) for emotion classification, and proved through experiments that HCNN is better at emotion recognition than traditional shallow models (such as SVM, KNN), especially for the recognition of Beta wave and Gamma wave.

### Cluster 5: Neurofeedback and Self-Regulation

Cluster 5 is the red part shown in Figure 5, which is named neurofeedback and self-regulation. Cluster 5 mainly includes brain computer interface, fMRI, real-time fMRI, neurofeedback, prefrontal cortex, self-regulation, emotions, virtual reality, children, etc.

Neurofeedback technology can feedback brain activities to individuals in real time, requiring individuals to autonomously regulate feedback signals, so as to realize self-management of brain activities and ultimately regulate individual behaviors (Sitaram et al., 2017). Neurofeedback is a channel for BCI to consciously access neural activity (Paret et al., 2018), which makes it possible to realize the automatic regulation of brain activity and has great application potential in enhancing and restoring emotion and cognitive (Lorenzetti et al., 2018).

Cluster 5 focuses on neurofeedback and self-regulation. Compared with the early EEG based neurofeedback technology, Cluster 1 pays more attention to the new real-time functional magnetic resonance imaging neurofeedback (rtfMRI-NFB) technology. This new neurofeedback technique requires participants to modulate brain activity during training by changing the oxygen-dependent signal index, which in turn achieves goals such as emotional and cognitive regulation (Herwig et al., 2019). Lorenzetti et al. (2018) proposed a multi-mode VR/rtfMR-NFB protocol as a brain activity intervention tool. It can provide virtual environments as BCI, play different music to stimulate users’ emotions and use personalized strategies to enhance the emotional intensity. Neurofeedback training is a clinical application of BCI and is widely used in children (such as children with developmental trauma and poor children) to regulate negative emotions, improve behavior, and ability. Rogel et al. (2020) suggested that neurofeedback training could reduce symptoms of post-traumatic stress disorder in children with developmental trauma and confirmed this idea using a randomized controlled study method. Antle et al. (2018) took poor children as the research object and proposed a neurofeedback system called Mind-Full to help poor children regulate negative emotions and focus their attention.

We use VOSviewer software to conduct the co-occurrence analysis of keywords, and analyze main research contents of each cluster in detail. Through reading articles on BCI and emotion research, we find that BCI is a communication mode that does not depend on peripheral nerves and muscles, but is completed by our brain to control the output of commands (Ash and Benson, 2018). The workflow of emotional brain computer interface generally includes five steps: emotion elicitation, signal acquisition, signal preprocessing, feature extraction, and classification, feedback and regulation. The five clusters we studied were divided according to the affective BCI process and the technology used.

Cluster 1 (External stimulus and ERP) corresponds to emotion elicitation, and the use of ERP can monitor people’s psychological responses to external stimulus. Cluster 2 (EEG and information collection) is related to the signal acquisition process, and EEG is used to record brain activity. Cluster 3 (SVM and information processing) and cluster 4 (Deep learning and emotion recognition) both correspond to information processing. Cluster 3 focuses on the application of SVM in processing EEG signals, and cluster 4 focuses on the application of deep learning in emotion recognition. Cluster 5 (Neurofeedback and self-regulation) is related to feedback and regulation process. Neurofeedback training can help subjects to autonomously regulate brain activities. The purpose of keyword cluster analysis is to construct the research framework of affective BCI and help readers better understand research contents in this field.

## Research Agenda

**FIGURE 6:**
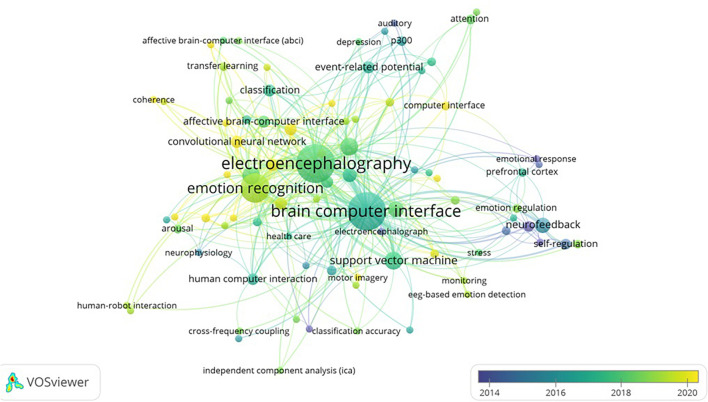
Map of research trends.

In this part, we use VOSviewer software to represent the time sequence of keywords by using graphical method. The color of the node represents the first time the keyword appeared, gradually transitioning from cool color to warm color. Blue represents the earliest year, and the research content is more classic. Yellow represents the latest year, and the research content is more cutting-edge. Figure 6 shows the time sequence of keywords in the 249 articles, which can help researchers understand research trends of BCI’s application in the field of emotion more clearly and intuitively.

By observing the newly emerging keywords and corresponding articles, we find that the existing researches are devoted to developing new technologies or new models to improve the accuracy of emotion recognition and have achieved good results, but there are still some shortcomings. For example, the article containing the keyword (transfer learning) hopes to solve the influence of individual differences on emotion recognition, because individual differences hinder the large-scale promotion of BCI. Although this problem has attracted the attention of scholars, there is still a lack of effective methods to solve this problem. This study proposes four future research directions, which will be elaborated below.

### Individual Differences Research

Due to the physiological differences between different users, for the same emotional stimulus, different individuals will induce different emotions, and physiological signals. The existence of individual differences creates a barrier to mass distribution of BCI. In order to solve the problem of individual differences, researchers use transfer learning to improve the generalization ability of emotional computing model (Wu et al., 2020). There are two methods of transfer learning: domain adaptation and domain generalization, both of which have their advantages and disadvantages. How to balance application domain adaptation and domain generalization to improve emotion recognition performance is a problem worth discussing. At present, there are still few studies on transfer learning, which is worth further study by scholars.

### The Extended Research of Brain-Computer Interface Application Scenarios

At present, BCI is mainly applied in medical field, game and entertainment field, learning and education field and military field (Vlek et al., 2012). With the increasing maturity of BCI technology, BCI will be applied to more fields in the future. In the future, social requirements for intelligent robots are becoming higher and higher, and BCI robots will have excellent development prospects. BCI smart home will be a direction for the future development of smart home. BCI smart home can meet needs of users more easily and conveniently, provided that BCI technology is mature enough. Existing studies have confirmed that using BCI to monitor users’ emotions in entertainment scenes such as music and movies can provide real-time and personalized services for users. In the future, with user permission, BCI can be applied to more entertainment scenarios.

### Privacy Risk Research

The protection of user information should be considered during the use of BCI. In the use of BCI, the disclosure of user information will lead to the illegal acquisition and utilization of users’ ideas, and even the disclosure of users’ thinking. For example, businesses may analyze users’ preferences and spending power based on their brain signals and induce unfair consumption (Jebari, 2013). Facing different application scenarios, privacy risks of BCI applications are different. We need to search possible countermeasures for different problems (Li et al., 2015). At present, researches on ethical issues in BCI have just begun. How to develop new technologies to protect user information is worthy of scholars’ attention and research (Agarwal et al., 2019). For different communication modes, such as brain to brain and brain to internet, future research needs to find new coping methods combined with BCI life cycle (Bernal et al., 2021).

### Others

The current application of BCI in the emotion field is limited to a certain technology or a certain model. The application of BCI should be a complete process, which requires researchers to conduct integrated research from the perspective of the whole process. Therefore, researchers should carry out more extensive cooperation and actively carry out interdisciplinary communication.

## Conclusion

In this study, literature related to affective BCI were collected and sorted out from WOS database. With the help of VOSviewer software, we conducted a scientific analysis of 249 literatures and systematically and comprehensively constructed the affective BCI research framework.

First, we found that the number of publications on emotional BCI is increasing year by year. Researches on affective BCI mainly focus on engineering, computer science, neurosciences neurology and psychology. Peoples R China, United States, and Germany have the largest number of publications. This study listed the top 10 authors with most publications and analyzed collaboration networks among authors. The top 10 most cited articles were also listed in this study.

Second, it is worth noting that we used the keyword co-occurrence function of VOSviewer software to conduct theme clustering analysis on keywords of 249 literatures. Clustering results were divided into 5 categories, which were named as external stimulus and ERP, EEG and information collection, SVM and information processing, deep learning and emotion recognition, neurofeedback and self-regulation, respectively. These five clusters are closely related to the BCI workflow.

Third, in the future research part, we pointed out individual differences research, privacy risk research, the extended research of BCI application scenarios and others deserve further study.

This study visually analyzes the application of BCI in the field of emotion and builds a framework for existing research. The results of this study reveal the shortcomings of current affective research based on BCI. The conclusions are helpful to guide the follow-up practitioners to actively use the new technology for emotion analysis.

## Data Availability Statement

The original contributions presented in the study are included in the article/supplementary material, further inquiries can be directed to the corresponding author/s.

## Author Contributions

All authors listed have made a substantial, direct and intellectual contribution to the work, and approved it for publication.

## Conflict of Interest

The authors declare that the research was conducted in the absence of any commercial or financial relationships that could be construed as a potential conflict of interest.

## Publisher’s Note

All claims expressed in this article are solely those of the authors and do not necessarily represent those of their affiliated organizations, or those of the publisher, the editors and the reviewers. Any product that may be evaluated in this article, or claim that may be made by its manufacturer, is not guaranteed or endorsed by the publisher.
